# Phenotype plasticity rather than repopulation from CD90/CK14+ cancer stem cells leads to cisplatin resistance of urothelial carcinoma cell lines

**DOI:** 10.1186/s13046-015-0259-x

**Published:** 2015-11-25

**Authors:** Margaretha A. Skowron, Günter Niegisch, Gerhard Fritz, Tanja Arent, Joep G. H. van Roermund, Andrea Romano, Peter Albers, Wolfgang A. Schulz, Michèle J. Hoffmann

**Affiliations:** Department of Urology, Medical Faculty, Heinrich-Heine-University Duesseldorf, Universitaetsstrasse 1, 40225 Düsseldorf, Germany; Institute of Toxicology, Medical Faculty, Heinrich-Heine-University Duesseldorf, Universitaetsstrasse 1, 40225 Düsseldorf, Germany; Department of Forensic Medicine, Medical Faculty, Heinrich-Heine-University Duesseldorf, Moorenstr. 5, 40225 Düsseldorf, Germany; Department of Urology, Maastricht University Medical Centre, P. Debyelaan 25, 6202 AZ Maastricht, The Netherlands; Department of Obstetrics and Gynaecology, GROW School for Oncology and Developmental Biology, Maastricht University Medical Centre, P. Debyelaan 25, 6202 AZ Maastricht, The Netherlands

**Keywords:** Urothelial carcinoma, Cancer stem cells, Cisplatin, CD90, CK14, Epithelial-mesenchymal-transition, WNT-signalling

## Abstract

**Background:**

Tumour heterogeneity and resistance to systemic treatment in urothelial carcinoma (UC) may arise from cancer stem cells (CSC). A recent model describes cellular differentiation states within UC based on corresponding expression of surface markers (CD) and cytokeratins (CK) with CD90 and CK14 positive cells representing the least differentiated and most tumourigenic population. Based on the fact that this population is postulated to constitute CSCs and the origin of cisplatin resistance, we enriched urothelial carcinoma cell lines (UCCs) for CD90 and studied the tumour-initiating potential of these separated cells in vitro.

**Methods:**

Magnetic- and fluorescence-activated- cell sorting were used for separation of CD90^+^ and CD90^−^ UCCs. Distribution of cell surface markers CD90, CD44, and CD49f and cytokeratins CK14, CK5, and CK20 as well as the effects of short- and long-term treatment with cisplatin were assessed in vitro and measured by qRT-PCR, immunocytochemistry, reporter assay and flow cytometry in 11 UCCs.

**Results:**

We observed cell populations with surface markers according to those reported in tumour xenografts. However, expression of cytokeratins did not concord regularly with that of the surface markers. In particular, expression of CD90 and CK14 diverged during enrichment of CD90^+^ cells by immunomagnetic sorting or following cisplatin treatment. Enriched CD90^+^ cells did not exhibit CSC-like characteristics like enhanced clonogenicity and cisplatin resistance. Moreover, selection of cisplatin-resistant sublines by long-term drug treatment did not result in enrichment of CD90^+^ cells. Rather, these sublines displayed significant phenotypic plasticity expressing EMT markers, an altered pattern of CKs, and WNT-pathway target genes.

**Conclusions:**

Our findings indicate that the correspondence between CD surface markers and cytokeratins reported in xenografts is not maintained in commonly used UCCs and that CD90 may not be a stable marker of CSC in UC. Moreover, UCCs cells are capable of substantial phenotypic plasticity that may significantly contribute to the emergence of cisplatin resistance.

**Electronic supplementary material:**

The online version of this article (doi:10.1186/s13046-015-0259-x) contains supplementary material, which is available to authorized users.

## Background

Bladder cancer (BC) is the 9th most common tumour world-wide and the most common cancer of the urinary tract [1]. In developed countries, about 90 % of BCs are urothelial carcinomas (UC) which may be further divided into two subgroups, as muscle-invasive and non-muscle-invasive. UCs are distinct in clinical behaviour and molecular alterations [2]. Muscle-invasive tumours, comprising up to one third of UC, frequently progress to metastatic disease and face a poor prognosis with only 50–60 % survival after 5 years [3]. In advanced and/or metastatic UC, platinum-based combination chemotherapy is the standard first-line treatment in the perioperative and the palliative setting [4, 5]. However, its impact on cancer-specific survival is limited. Despite frequent initial treatment responses, overall survival does not exceed 12–16 months in metastatic patients [6].

Resistance to cisplatin in UC is brought about by a variety of mechanisms [7]. In particular, resistance may emerge via repopulation by cancer stem cells (CSC) [8, 9]. According to this model, while significantly reducing cancer cell burden, cisplatin treatment indirectly stimulates CSC to promote cancer recurrence. Hence, targeting these cells would increase the efficacy of platinum-based chemotherapy. However, it is contentious how to define CSC populations in UC and they could differ between individual cases [10, 11]. Generally, CSCs are thought to possess self-renewal potential, to reversibly enter quiescent or dormant states, and to be more resistant to cytotoxic drugs, thereby contributing to therapeutic resistance [9, 12–14]. To which extent these properties apply to CSC in UC is unknown.

In addition to CSC, urothelial tissue hierarchy and the mechanisms regulating its normal or aberrant differentiation are incompletely understood. Previously, all cell types of the normal urothelium were thought to originate from the least differentiated basal cells, positive for both cytokeratin 5 (CK5) and cluster-of-differentiation-44 (CD44) [15, 16]. CK14 is a further marker for basal cells which is rarely detectable in normal urothelium, but significantly expressed in cells with squamous differentiation [17]. Intermediate cells and terminally differentiated umbrella cells expressing uroplakins and CK20 were thought to develop from basal cells. However, new data argues for a second urothelial lineage producing intermediate and umbrella cells [18, 19]. Thus, the cells of origin of UC remain unidentified. Numerous attempts to isolate CSCs from UC tumour tissues have yielded heterogeneous marker profiles [20–22].

**Fig. 1:**
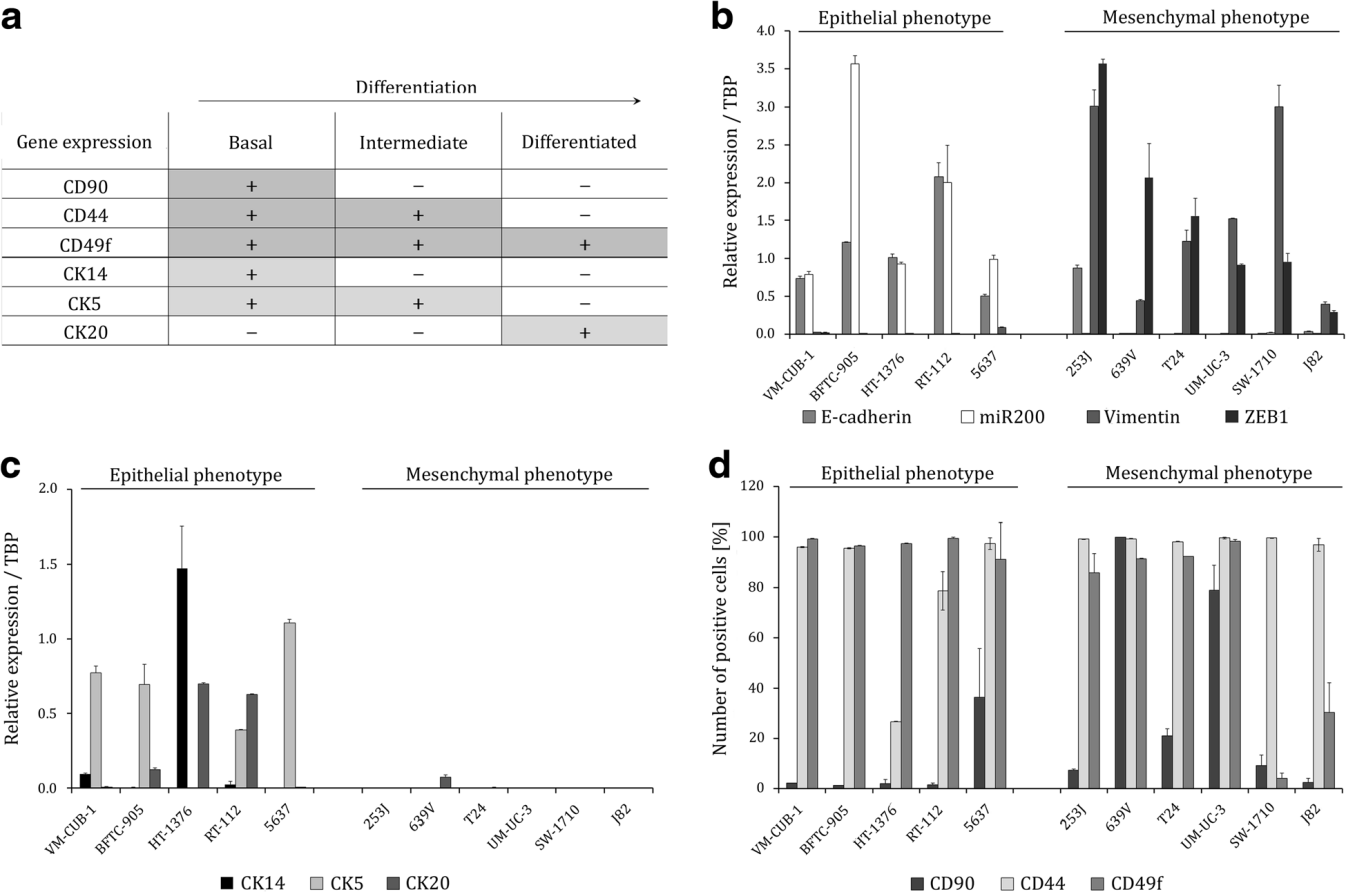
UCCs are heterogeneous for cytokeratin expression and proportions of differentiation states. **a** Differentiation state model of UC according to Volkmer et al. [10]. Relative mRNA expression of epithelial markers *E-cadherin* and *miR-200* and mesenchymal markers *Vimentin* and *ZEB1* (**b**) and *CK14*, *CK5*, and *CK20* (**c**) measured by qRT-PCR in a panel of 11 human UCCs. UCC expression levels were quantified relative to an internal standard. *TBP* was used as reference gene. **d** Mean percentages of CD90, CD44, and CD49f positive cells in 11 UCCs as measured by flow cytometry. UCCs were categorized into epithelial and mesenchymal phenotype. Values are expressed as the mean ± SD of triplicates

In studies aiming at defining molecular subtypes of UC, *de novo* expression of CK14 in a so-called ‘basal’ subtype was generally indicative of unfavourable prognosis [10, 20, 22], suggesting that a subpopulation of less differentiated, CK14-positive cells might drive an aggressive type of UC. Further, *in silico* analysis of expression data and xenograft experiments using primary patient-derived cells led has to a hierarchical ‘differentiation state’ model for UC [10]. In this model, cellular subpopulations within primary UC tumours were assigned to ‘differentiation states’ according to a correlated expression profile of cytokeratins (CK14, CK5, CK20) and surface markers (CD90, CD44, CD49f) (Fig. 1a). CD90 and CK14 double positive cells were the least differentiated cell type in primary UC specimens and were highly tumourigenic in xenograft experiments, implicating CD90 and CK14 as markers of a CSC population in UC. Of note, the abundance of subpopulations was also heterogeneous in primary tumours and CD90-positive cells could not be isolated from every patient. In such cases, the next least differentiated subpopulation in the postulated hierarchy proved to be tumourigenic in xenografts. Unfortunately, such cell populations were not further phenotypically characterized regarding stemness or cisplatin resistance due to limited material from primary tissues. Thus, we wondered whether this model also holds for established UC cell lines (UCCs), which are commonly used as models of the disease [23] and allow detailed characterization of cellular properties and differentiation hierarchies.

To this end, we determined the abundance of CK14/CD90-positive cells in UCCs and investigated whether they possess stem cell-like properties and are more resistant against treatment with cisplatin. In detail, we determined expression levels and distribution of CD90, CD44, and CD49f as well as CK14, CK5, and CK20 in a panel of 11 UCCs representing various subtypes, stages, and grades of the disease. Further, we examined the correlation between CD90 and CK14 expression and analysed clonogenic and proliferative potential as well as cisplatin sensitivity of CD90^+^ cells after immunomagnetic enrichment and flow cytometry-based sorting. In addition, we evaluated whether short-term or long-term treatment with cisplatin enriched for CD90-positive cells.

## Methods

### Cell culture, treatment, and transfection

The human UC cell lines RT-112, VM-CUB-1, UM-UC-3, T24, 639 V, 253 J, 5637, SW170, HT-1376, BFTC-905, and J82, kindly provided by M. A. Knowles (Leeds, UK), J. Fogh (New York, NY), B. Grossmann (Houston, TX), or the DSMZ (Braunschweig, Germany), were grown in DMEM GlutaMAX-I (Gibco, Darmstadt, Germany) containing 10 % fetal calf serum. All cell lines were recently verified by standard DNA fingerprint analysis. For short-term experiments a single dose of cis-diamminedichloroplatinum-II (cisplatin; Accord Healthcare, London, UK) was added for 72 h; long-term treated (LTT) UCCs were generated by adding cisplatin after every passage at escalating doses over 8–10 months. Niclosamide was purchased from Sigma-Aldrich (#N3510, St. Louis, MO) and dissolved in DMSO. Reporter plasmids TopFlash or FopFlash (Merck Millipore, Billerica, MA) and mutated β-catenin-S33Y, kindly provided by H. Clevers (Utrecht, The Netherlands), were transfected using X-tremeGENE9 DNA Transfection Reagent (Roche, Basel, Switzerland) according to the manufacturer’s instructions.

### RNA extraction, cDNA synthesis, and quantitative real-time PCR

RNA was prepared using QIAzol Lysis Reagent (Qiagen, Hilden, Germany) and RNeasy Mini Kit (Qiagen, Hilden, Germany) according to the manufacturer′s instructions. cDNA-synthesis was performed using the QuantiTect Reverse Transcription Kit (Qiagen, Hilden, Germany). QuantiTect SYBR Green (Qiagen, Hilden, Germany) was used for quantitative real-time PCR. Gene expression levels were determined using self-designed primers on the Lightcycler 2.0 Instrument (Roche, Basel, Switzerland) (Additional file 1: Table S1). The housekeeping gene *TBP* was routinely used for normalization of marker gene expression results. As *TBP* levels appeared to be slightly affected by treatment with cisplatin, we used the housekeeping gene *SDHA* for normalizing data of some experiments.

### Measurement of cell viability and proliferation

Cell viability was generally measured in quadruplicates by means of MTT assay (Sigma-Aldrich, St. Louis, MO). For the reason of increased sensitivity the CellTiter-Glo-assay (Promega, Fitchburg, WI) was used to analyse rare cells from FACS sorting experiments.

### Immunofluorescence

UCCs were fixed on coverslips with 4 % formaldehyde. Cells were permeabilized at room temperature for 10 min with ice-cold methanol, 30 min with 0.1 % saponin, and 3 min with 0.5 % Triton-X-100 for E-cadherin, Vimentin, and β-catenin staining, respectively. Subsequent to blocking, UCCs were incubated with primary antibodies (E-cadherin #3195, Cell Signaling, Cambridge, UK, 1:100; CD90 ab133350, Abcam, Cambridge, UK, 1:200; CK14, Clone CKB1, Sigma-Aldrich, St. Louis, MO, 1:50; Vimentin, ab92547, Abcam, Cambridge, UK, 1:400; β-catenin, #9562, Cell Signaling, Cambridge, UK, 1:50). Secondary antibodies (Life Technologies, Carlsbad, CA) were added for 1 h in the dark at room-temperature. Cover slips were counter-stained with 0.5 mg/ml DAPI.

### Clonogenicity and sphere formation assay

CD90^+^ and CD90^−^ cells were seeded after magnetic or FACS sorting into 6 cm plates at a density of 3 × 10^3^ cells per well. Visible clones were consecutively fixed in 50 and 100 % methanol before Giemsa staining (Merck, Darmstadt, Germany). Sphere formation was analysed subsequent to FACS sorting and automated deposition of single cells.

### Cell separation

For CD90 separation, 15 × 10^6^ cells were labelled with CD90-PE antibody and captured by means of anti-PE microbeads and a magnetic LS-column according to the manufacturer’s protocol (Miltenyi Biotec GmbH, Bergisch Gladbach, Germany). FACS sorting was performed by a MoFlo XDP with Summit 5.3 software (Beckman Coulter, Brea, CA).

### Flow cytometry

Expression of cell surface markers CD90, CD44, and CD49f was determined using a MACSQuant flow cytometer using the MACSQuant Analyzer 10 software (Miltenyi Biotec GmbH, Bergisch Gladbach, Germany) and labelled antibodies CD90-PE, CD44-APC, and CD49f-FITC (Miltenyi Biotec GmbH, Bergisch Gladbach, Germany).

### Statistical analysis

Statistical analysis was performed with SPSS Statistics software version 21 (IBM, Armonk, NY). Differences between groups were analysed using Student’s *t*-test after checking for normal distribution of results. All differences highlighted by asterisks were statistically significant and are encoded in figures as *P* <0.05 * or *P* <0.001 **.

## Results

### UCCs are heterogeneous for cytokeratin expression and proportions of differentiation states

First, we characterized a representative panel of 11 UCCs for the expression of markers associated with cellular morphology (mesenchymal-like vs. epithelial-like), cytokeratins (CKs) CK14, CK5, and CK20 and cluster-of-differentiation (CD) surface markers CD90, CD44, and CD49f. According to the recently published hierarchical ‘differentiation state model’ for UC the correlated expression of both CK and CD markers may be used to assign cellular subpopulations to three defined differentiation states (Fig. 1a) [10]. Differences in morphology among UCCs were also reflected distinctively on the molecular level. As expected, epithelial markers (*E-cadherin, miR200*) were almost exclusively expressed in cell lines with epithelial morphology (VM-CUB-1, BFTC-905, HT-1376, RT-112, 5637). In contrast, mesenchymal markers (*Vimentin*, *ZEB1*) were almost undetectable in UCCs with an epithelial phenotype, but strongly expressed in UCCs with a mesenchymal phenotype (253 J, 639 V, T24, UM-UC-3, SW-1710, J82; Fig. 1b). Surprisingly, expression analysis of cytokeratins revealed that mesenchymal phenotype UCCs lacked RNA-expression of CK14, CK5, and CK20 (Fig. 1c). In addition, cytokeratin expression differed also among epithelial UCCs. Whereas the CK14 expression level in VM-CUB-1 cells resembled that of normal cultured uroepithelial cells from ureters (data not shown), expression was 14-fold higher in HT-1376 cells and 5-fold lower in RT-112. CK5 was robustly expressed in all epithelial-like UCCs, with the exception of HT-1376, and CK20 was detectable in BFTC-905, HT-1376, and RT-112.

As expected, we observed a heterogeneous distribution of the cell surface markers CD90, CD44, and CD49f by flow cytometry among UCCs. In contrast to cytokeratins, surface markers were generally expressed independently of the morphological phenotype (Fig. 1d, Additional file 2: Figure S2). In many UCCs only a small number of cells were positive for CD90, whereas other lines contained significant numbers of CD90-positive cells, so that their abundance varied between 0.75 and 99.5 % across the panel. For example, the 639 V cell line appeared to contain exclusively triple-positive (i.e. CD90^+^ CD44^+^ CD49f^+^) cells. Intriguingly, this cell line did not express CK14 at detectable levels. With the exception of HT-1376, all cell lines contained a high fraction of CD44^+^ cells. Similarly, almost all UCCs were CD49f^+^, only the cell lines SW-1710 and J82 contained few CD49f^+^ cells (Fig. 1d*,* Additional file 2: Figure S2). Taken together, this characterization demonstrated that UCCs collectively reflect the heterogeneity observed among primary UCs, but also indicated that the proposed correlation between CK and CD markers does not apply to UCCs, especially in mesenchymal cell lines lacking cytokeratins.

### Enrichment of CD90^+^ cells does not enrich for CK14 expression in UCCs

**Fig. 2:**
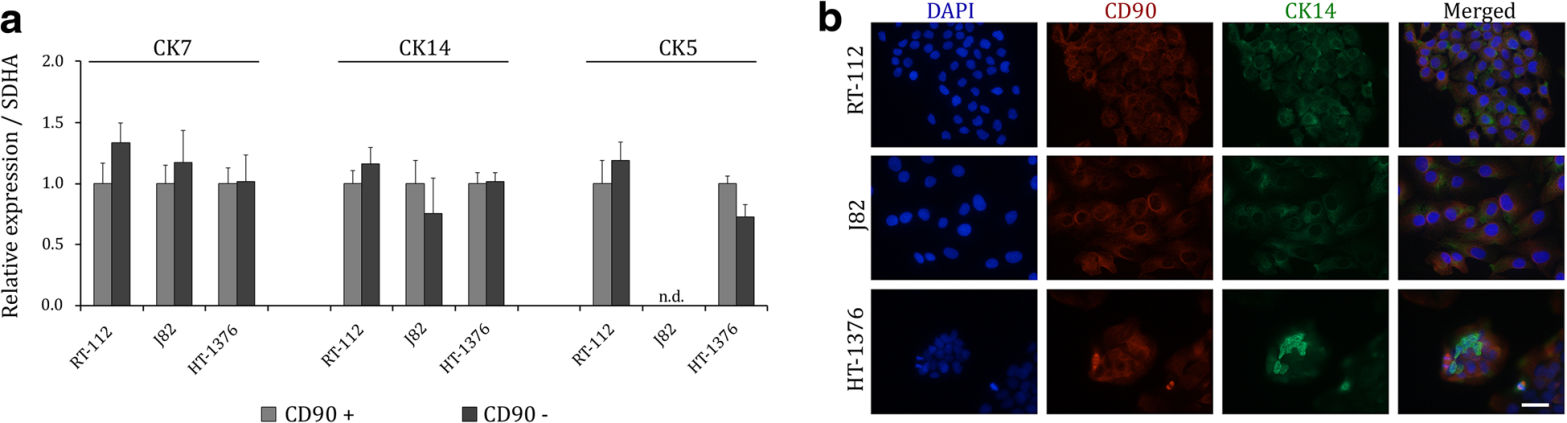
Enrichment of CD90^+^ cells does not enrich for CK14 expression in UCCs. **a** Relative RNA expression of *CK7*, *CK14*, and *CK5* in RT-112, J82, and HT-1376 cells magnetically enriched for CD90. Expression levels of the CD90^+^ cell fraction were set as 1. *nd* not detectable. **b** Representative immunofluorescence stainings of CD90 (*red*) and CK14 (*green*) for cell lines RT-112, J82, and HT-1376; DAPI was used for nuclear staining. Scale bars, 50 μm. Values are expressed as the mean ± SD of triplicates

**Fig. 3:**
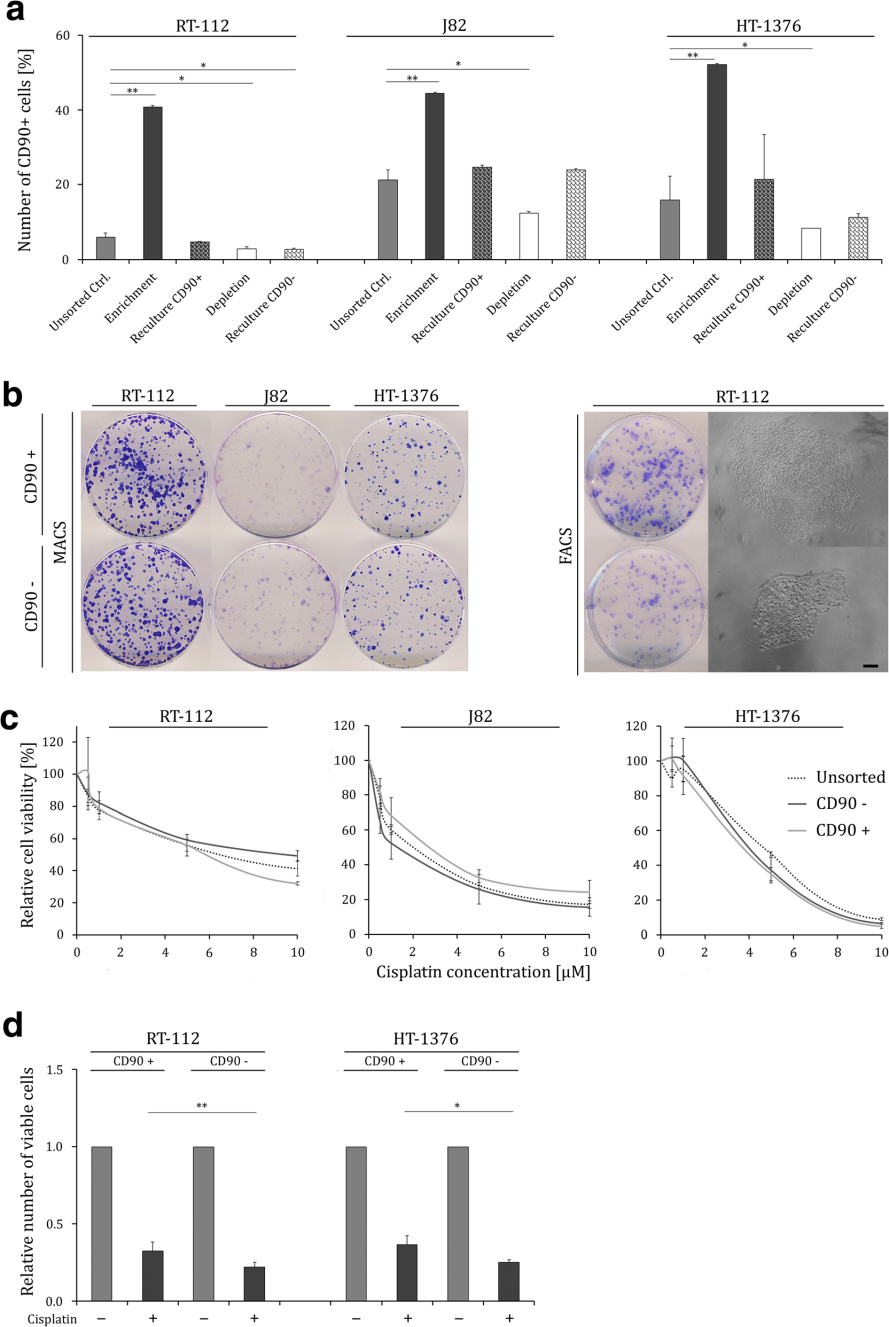
CD90^+^ UCCs do not exhibit a distinct stem cell-like phenotype. **a** CD90^+^ fraction in unsorted (*grey bars*), CD90 enriched (*dark grey bars*), CD90 enriched cells after reculturing for about 7–8 population doublings (*dark grey bars, shaded*), CD90 depleted (*white bars*), and CD90 depleted cells after reculturing (*white bars, shaded*) in RT-112, J82, and HT-1376 as measured by flow cytometry. **b** Clonogenic potential in magnetically and FACS sorted CD90^+^ and CD90^−^ populations from RT-112, J82, and HT-1376 cell lines shown by Giemsa staining. Colony-forming potential of single cells positive or negative for CD90 from RT-112 cells after FACS sorting. Phase-contrast microscopy, scale bars, 100 μm. **c** Cisplatin sensitivity was measured in unsorted and MACS sorted CD90^+^ and CD90^−^ fractions of RT-112, J82, and HT-1376 by MTT assay after 72 h treatment. Untreated cells were set as 100. **d** Relative cisplatin sensitivity of FACS sorted CD90^+^ and CD90^−^ cells from RT-112 and HT-1376 cell lines as measured by CellTiter-Glo Luminescent Cell Viability Assay after 72 h cisplatin treatment with IC_50_ concentrations (see Fig. 4a). Untreated cells were set as 1. Values represent the mean ± SD of quadruplicates. *P <0.05; **P <0.001

We further investigated the postulated correlation between CD90 and CK14 by enrichment of CD90-positive cells and subsequent expression analysis of cytokeratins. To this end, we selected three cell lines representative for different epithelial-like or mesenchymal-like phenotypes, with variable expression of CD90 and CK14 (RT-112: epithelial, CD90^low^, CK14^intermediate^; J82: mesenchymal, CD90^low^, CK14^low^; HT-1376: epithelial, CD90^low^, CK14^high^). The efficiency of immunomagnetic enrichment of CD90^+^ cells was monitored by triple staining (CD90, CD44, CD49f) via flow cytometry. After enrichment the abundance of CD90-positive cells was significantly increased up to 50 % (Fig. 3a, Additional file 3: Figure S1a). Subsequently, mRNA expression of *CK14, CK5,* and *CK7,* which is known to be robustly expressed in all uroepithelial cells, was quantified by qRT-PCR (Fig. 2a). As expected, the pan-urothelial cytokeratin *CK7* did not differ significantly between the populations. However, *CK14* expression, too, was not significantly differentially expressed between CD90^+^ and CD90^−^ cell fractions. *CK5*, a marker of basal and intermediate differentiation states in UC (Fig. 2a), was invariable in RT-112, but decreased in the CD90^−^ fraction of HT-1376. Concurring with the analysis above (Fig. 1c), *CK5* expression was below the limit of detection in J82 cells (Fig. 2a). Accordingly, immunofluorescence staining for CD90 and CK14 demonstrated that both markers were heterogeneously expressed in the cell lines, but not generally co-expressed, as illustrated for the HT-1376 cell line in Fig. 2b.

### CD90^+^ UCCs do not exhibit a distinct stem cell-like phenotype

Next, we investigated whether CD90^+^ cells isolated from UCCs exhibit CSC properties by measuring clonogenicity, self-renewal and differentiation capacity or increased resistance to cisplatin. Following magnetically CD90 enrichment or depletion, CD90^+^ and CD90^−^ subpopulations of RT-112, J82 and, HT-1376 were recultured and followed over time. After reculturing, the number of CD90^+^ cells in enriched cultures regressed to baseline expression. Interestingly, in the CD90 depleted subpopulation, the amount of CD90^+^ cells likewise increased to the baseline level (Fig. 3a*,* Additional file 3: Figure S1b). Similarly, we did not observe any difference in colony forming potential between CD90 enriched and CD90 depleted cell fractions subsequent to immunomagnetic sorting (Fig. 3b; MACS panel). Since immunomagnetic sorting only allows enrichment but not complete separation of cell fractions, RT-112 and HT-1376 cell lines were additionally sorted by flow cytometry. Colony formation assays of highly purified cell fractions revealed a slight advantage for CD90^+^ cells (Fig. 3b; FACS panel). We also analysed self-renewal capacity subsequent to FACS sorting by seeding single cells in 96-well-plates. Although spheres from CD90^+^ cells appeared to grow slightly faster (Fig. 3b; right panel) we did not observe a significant difference in the number of colonies originating from single CD90^+^ or CD90^−^ RT-112 cells. Thus, RT-112 CD90^−^ cells as well exhibited self-renewal capacity. HT-1376 cells did not form colonies after single cell isolation (data not shown).

Additionally, CD90^+^ magnetically enriched and FACS sorted cell fractions were examined for their cisplatin sensitivity. CD90^+^ enriched cell fractions from RT-112, J82, and HT-1376 cell lines were no more resistant to cisplatin than the corresponding CD90 depleted fractions (Fig. 3c). However, highly purified FACS sorted CD90^+^ cells were less sensitive to cisplatin treatment than CD90^−^ cells from RT-112 and J82 cell lines (Fig. 3d).

### UCCs sensitivity towards short-term treatment with cisplatin is not correlated with abundance of CD90^+^ cells

**Fig. 4:**
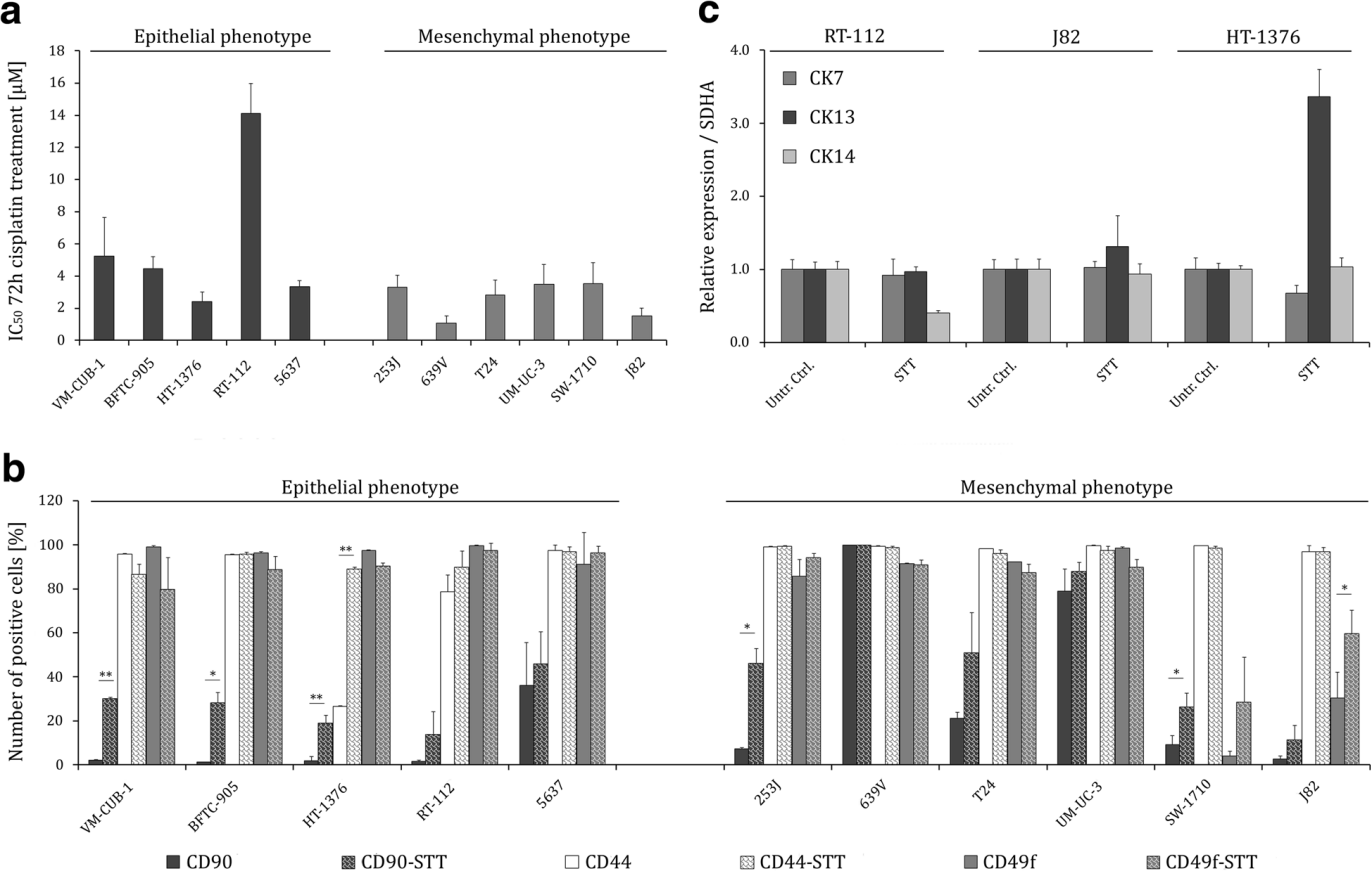
UCCs sensitivity towards short-term treatment with cisplatin is not correlated with abundance of CD90^+^ cells. **a** Cell viability was measured 72 h after cisplatin treatment by MTT assay in 11 UCCs, categorized into UCCs with epithelial (*dark grey bars*) and mesenchymal (*light grey bars*) phenotypes. **b** Subsequent to short-term treatment with cisplatin (STT, 72 h) most cell lines displayed increased numbers of CD90+ cells. Mean percentages of CD90^+^ cells in untreated (*dark grey bars*) and STT (*dark grey bars, shades*), CD44^+^ cells in untreated (*light grey bars*) and STT (*light grey bars, shades*), and CD49f^+^ cells in untreated (*grey bars*) and STT (*grey bars, shades*) UCCs as measured by flow cytometry. c) Relative expression of *CK14*, *CK5*, and *CK20* was measured by qRT-PCR in a panel of 11 untreated and STT-UCCs. Expression in the respective untreated control cells was set as 1. Data represent the mean ± SD of three independent experiments. *SDHA* was used as reference gene and relative expression calculated by using the 2^−ΔΔCT^ method. *Untr Ctrl* untreated control, *STT* short-term cisplatin treatment. *P <0.05; **P <0.001

To investigate the relation between the abundance of CD90^+^ cells and cisplatin sensitivity, we sought to identify the appropriate doses and time schedule for cisplatin treatment. Thus, we determined IC_50_ concentrations for cisplatin after 48 and 72 h and also checked for changes in the abundance of CD90^+^ cells from 24 to 96 h by flow cytometry (Fig. 4a). Based on the results, the following experiments were performed within a period of 72 h, IC_50_ values ranged between 1.07 and 12.5 μM (Fig. 4a*)*. Across the cell lines, no correlation was obvious between the abundance of CD90^+^ cells (Fig. 1d) and sensitivity to cisplatin (Fig. 4a). For instance, the 639 V cell line comprising the biggest fraction of CD90^+^ cells was highly sensitive to cisplatin, whereas RT-112 containing a small fraction of CD90^+^ cells was the most resistant cell line. However, following short-term treatment (STT) with cisplatin at IC_50_ doses for 72 h, the abundance of CD90^+^ cells increased significantly in 6/11 UCCs, particularly in cell lines with originally low abundance of CD90^+^ cells (Fig. 4b*,* Additional file 2: Figure S2). The fraction of CD44^+^ and CD49f^+^ cells was augmented only in UCCs with low abundance of these markers, namely HT-1376 (CD44^low^) and J82 (CD49f^low^).

We additionally analysed the cell lines with an increased fraction of CD90^+^ cells after cisplatin treatment for cytokeratin RNA expression. Again, cytokeratins generally expressed in uroepithelial cells, like CK7 and CK13, remained mostly unchanged and no increase in *CK14* expression was observed (Fig. 4c).

### Long-term cisplatin treated UCCs are not enriched for CD90^+^/CK14^+^ cells

**Fig. 5:**
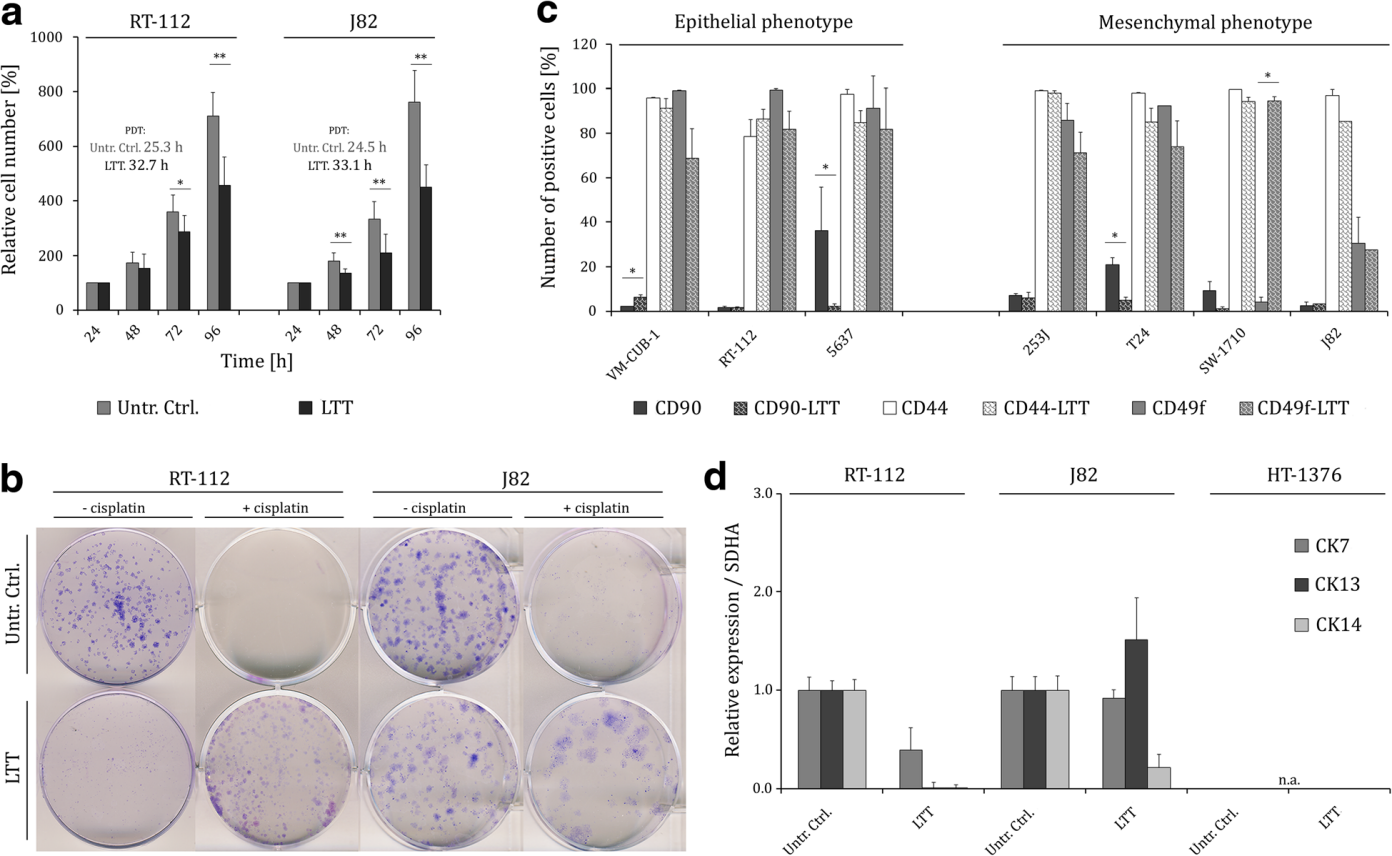
Long-term cisplatin treated UCCs are not enriched for CD90^+^/CK14^+^ cells. **a** Relative cell number in RT-112-LTT and J82-LTT and parental cell lines was measured by MTT after 24, 48, 72 and 96 h. Population doubling time was calculated based on raw absorbance data. **b** Clonogenic potential of RT-112-LTT and J82-LTT and parental cell lines without or with cisplatin, Giemsa staining. **c** Mean percentages of CD90^+^ cells in untreated (*dark grey bars*) and LTT (*dark grey bars, shades*), CD44^+^ cells in untreated (*light grey bars*) and LTT (*light grey bars, shaded*), and CD49f^+^ cells in untreated (*grey bars*) and LTT (*grey bars, shaded*) UCCs as measured by flow cytometry. **d** Relative expression of *CK7*, *CK13*, and *CK14* in untreated and LTT-UCCs. Expression levels in the untreated control were set as 1. *SDHA* was used as a reference gene and relative expression was calculated by the 2^−ΔΔCT^ method. Values represent the mean ± SD of biological triplicates. *Untr Ctrl* untreated control, *LTT* long-term cisplatin treatment, *PDT* population doubling time, *na* not available. *P <0.05; **P <0.001

**Fig. 6:**
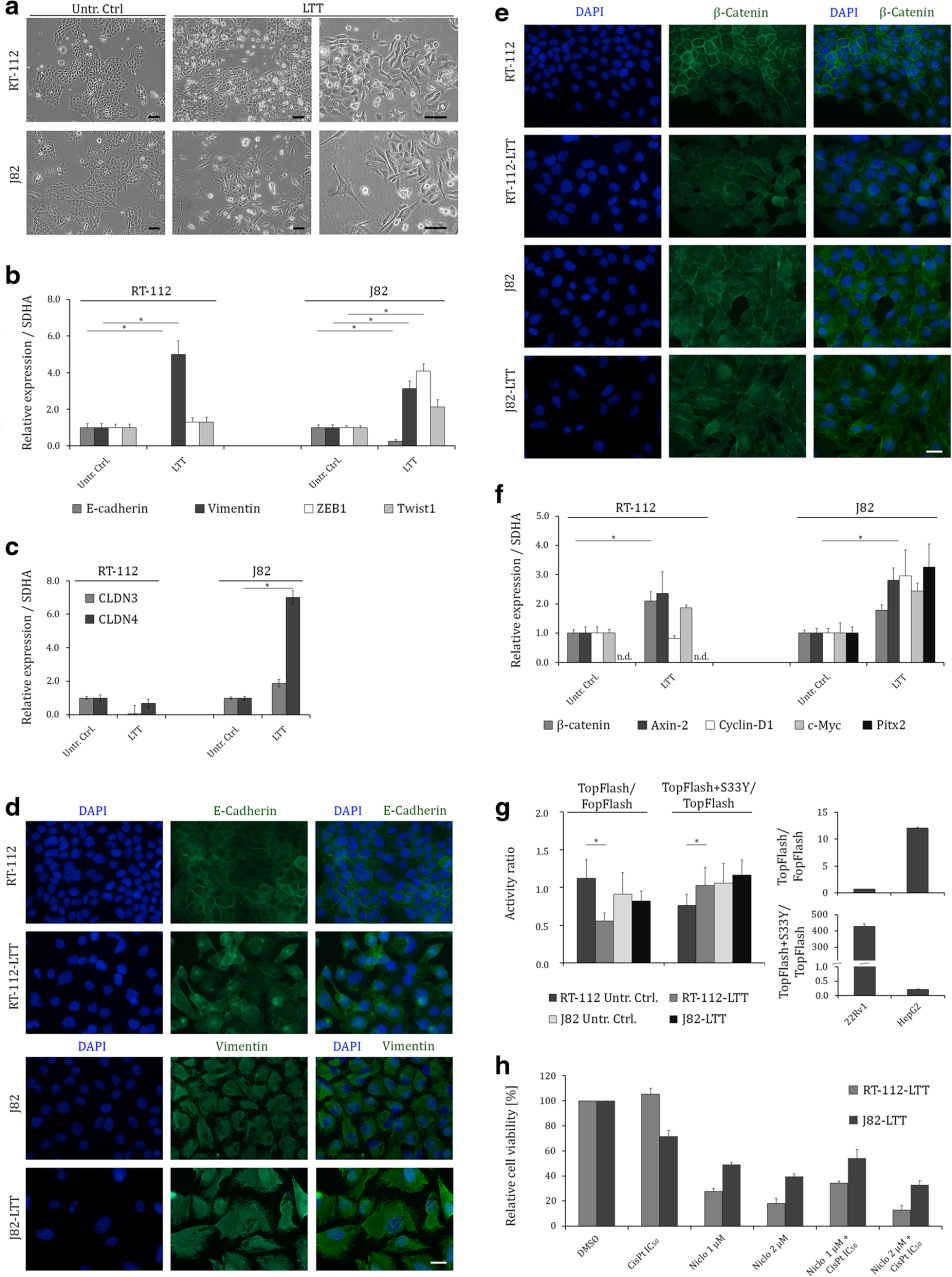
Activation of WNT-signalling may contribute to survival of UCCs upon long-term cisplatin treatment. **a** Morphology of RT-112-LTT and J82-LTT and their parental cell lines. Scale bars, 100 μm. qRT-PCR demonstrated relative expression levels of *E-Cadherin*, *Vimentin*, *Twist1*, *ZEB1* (**b**) and *CLDN3* and *CLDN4* (**c**) in RT-112-LTT and J82-LTT and their parental cell lines. **d** Immunofluorescence stainings for E-cadherin and Vimentin (**d**) and β-Catenin (**e**) in RT-112-LTT and J82-LTT and their parental cell lines. DAPI staining (*blue*) was used to visualize nuclei. Scale bars, 50 μm. **f** Relative RNA expression levels of *β-Catenin*, *AXIN-2*, *CCDN1*, *c-MYC,* and *PITX2* in untreated and RT-112-LTT and J82-LTT. Expression levels in the untreated control were set as 1. **g** Basal and inducible activity of a TCF/β-Catenin-dependent promotor. Mean ± SD of duplicates of TopFlash/FopFlash and TopFlash+S33Y/TopFlash ratio are shown in RT-112-LTT and J82-LTT and their parental cell lines. 22RV1 and HepG2 cell lines were used as controls. **h** Relative cell viability was measured 72 h after treatment with cisplatin, niclosamide, or combination of both by MTT assay in RT-112-LTT and J82-LTT. *P <0.05; **P <0.001

Since some enrichment of CD90^+^ cells was observed after short-term treatment with cisplatin, we wondered whether long-term cisplatin treatment further selects for this cell fraction. RT-112, J82, and HT-1376 cells underwent long-term cisplatin treatment (LTT) over several months with escalating doses, following a protocol similar to the Resistant Cancer Cell Line (RCCL) collection [24]. Ultimately, resistant RT-112 and J82 cells could be maintained as proliferating cultures with 50 and 1.6 μM cisplatin, respectively, added after each passage. No long-term surviving cells could be obtained from HT-1376. IC_50,_ determined after 72 h treatment of LTT cell lines, increased significantly compared to the corresponding parental cells. In RT-112-LTT IC_50_ for cisplatin was >50 μM compared to 12.5 ± 2.7 μM in the parental cells and IC_50_ for J82-LTT increased from 1.5 ± 0.48 μM to 9.2 ± 4.2 μM. The RT-112-LTT cells grew much slower than the parental line, with population doubling times of 32.7 and 25.3 h, respectively. Similar results were found in J82-LTT and untreated parental J82 with doubling times of 33.1 and 24.5 h, respectively (Fig. 5a). Moreover, the LTT-UCCs were much less clonogenic than the parental cells in the absence of cisplatin (Fig. 5b). However, upon cisplatin treatment, only LTT-UCCs were capable of clonogenic growth compared to the parental controls indicating their growth advantage through acquired cisplatin resistance (Fig. 5b). Subsequently, CD90-, CD44- and, CD49f-positive cell fractions as well as mRNA expression of CK7, CK13, and CK14 were determined in theRT-112-LTT and J82-LTT. In contrast to the STT, CD90^+^ cells were not enriched in LTT sublines (Fig. 5c*,* Additional file 4: Figure S3). The fraction of CD44 and CD49 positive cells also remained largely unchanged as compared to the parental cell lines. To corroborate our results on resistant RT-112 and J82 cells, further resistant UCCs were generated (Fig. 5c, Additional file 4: Figure S3; VM-CUB-1-LTT, 5637-LTT, 253 J-LTT, T24-LTT, SW-1710-LTT). In these cell lines, too, CD90^+^ cells were not enriched compared to their parental UCC. On the contrary, a significant decrease of CD90^+^ cells was observed in 5637-LTT and T24-LTT. Expression of CK14 was not increased in any LTT-cell line, rather expression of cytokeratins decreased generally (Fig. 5d). Fitting the decreased expression of cytokeratins, RT-112-LTT and J82-LTT cells underwent morphological changes. While parental RT-112 cells form epithelial colonies with strong cell-to-cell-adhesion, RT-112-LTT colonies were less compact and cells often assumed a more mesenchymal phenotype (Fig. 6a). Compared to the untreated controls, RT-112-LTT and J82-LTT increased in size. In summary, morphological changes pointed towards an epithelial to mesenchymal transition (EMT) in UCCs upon LTT.

### Phenotypic plasticity of UCCs facilitates evasion from long-term treatment with cisplatin

To verify that UCCs undergo EMT upon long-term treatment with cisplatin, we compared the mRNA levels of *E-cadherin*, *Vimentin*, *ZEB1*, and *Twist1* between paternal and LTT-cells (Fig. 6b). In accord with the morphological changes, expression of the epithelial marker *E-cadherin* decreased significantly in RT-112-LTT and J82-LTT whereas expression of the mesenchymal marker *Vimentin* increased significantly. Likewise, the expression of transcription factors inducing EMT, such as *ZEB1* and *Twist1*, was increased in the LTT-lines, albeit not significantly in RT-112-LTT. As we observed less tight cell-cell-contacts after cisplatin treatment, we also quantified the expression of *CLDN3* and *CLDN4*, encoding structural molecules of tight junctions. Indeed, *CLDN3* and *CLDN4* expression was decreased in RT-112-LTT (Fig. 6c), derived from an epithelial cell line, but not in in J82-LTT, derived from a cell line with mesenchymal phenotype. IF staining for E-cadherin in RT-112-LTT and Vimentin in J82-LTT confirmed the mRNA results (Fig. 6b, d). Further, IF staining for β-catenin revealed diminished membrane localisation, but no major shift into the nucleus (Fig. 6e).

### Activation of WNT-signalling may contribute to survival of UCCs upon long-term cisplatin treatment

As some results pointed towards an activation of canonical WNT-signalling in LTT-cells, we measured downstream targets of the pathway, namely *AXIN-2*, *CCDN1*, *c-MYC*, and *PITX2*. Indeed, the mRNA expression of these genes was increased in LTT-UCCs compared to their parental cell lines (Fig. 6f). We further confirmed augmented WNT-pathway activity by the TOP-FOPflash-assay indicating that the pathway could be activated by transfection of mutant β-catenin-S33Y in RT-112-LTT, but not in parental cells (Fig. 6g) [25]. Positive controls for endogenous and inducible β-catenin activity in HepG2 and 22Rv1 cell lines, respectively, demonstrated the functionality of the assay (Fig. 6g). Next, we investigated whether treatment of LTT sublines with the WNT-inhibitor niclosamide might revert cisplatin resistance of the cells. To this end we performed a dose response curve for niclosamide after 72 h treatment (Additional file 5: Figure S4a). We used the colorectal carcinoma cell line HCT-116 as a control for a cell line with significant WNT-pathway activity [26]. HCT-116 cells were more sensitive towards niclosamide treatment than RT-112-LTT and J82-LTT, as well as their parental cell lines (Additional file 5: Figure S4a, b). As expected, treatment of HCT-116 cells with niclosamide for 72 h at IC_50_ resulted in downregulation of the WNT/β-catenin targets β-catenin, Axin-2, and CyclinD1 (Additional file 5: Figure S4c upper panel; *p* <0.05). We further demonstrated that WNT-signalling activity could be significantly inhibited in HCT-116 cells by the applied doses of the compound in the TOP-FOPflash-reporter assay (Additional file 5: Figure S4d). Thus, the compound is active at the applied concentrations in cell lines with canonical activation of the WNT/β-catenin signalling. However, we did not observe synergistic effects on cell viability of resistant LTT cell lines by combined treatment with cisplatin and niclosamide (Fig. 6h) indicating that cisplatin resistance could not be reverted by this inhibitor. Moreover, treatment with niclosamide did not result in significant expression changes of WNT- target genes UC cells (Additional file 5: Figure S4c, lower panel).

## Discussion

Since drug resistance remains a major limitation to UC chemotherapy [9], a better understanding of mechanisms underlying both, inherent or acquired resistance, appears mandatory to overcome the current stagnation in therapeutic efficiency. Inherent drug resistance may originate from CSC endowed with self-renewal capacity and inherent chemoresistance [27, 28]. However, the identity of CSC in UC has not been clearly defined. Accordingly, a better understanding of the cell lineages in benign and cancerous urothelial tissues is urgently needed. Likewise, the identification of specific markers for isolation of individual cell populations will be a prerequisite for a comprehensive characterization of their cellular properties.

In the past, a variety of markers, which had mainly been developed from studies of normal adult stem cells [29], have been used to identify CSC in solid tumours, leading to heterogeneous or sometimes contradictory results [30]. The issue is complicated by tumour heterogeneity and by the possibility that tumours might contain more than one distinct CSC population [31]. Putative CSCs have been isolated from many tumour types including leukaemia, melanoma, and various carcinomas using markers such as CD133, CD24, CD44, and ALDH1 [32, 33]. Obviously, markers suitable for one cancer type may not be universally applicable. For example, CD24 is frequently used to isolate CSCs from solid tumours, e.g. breast cancer, but does not enrich tumour-initiating cells from UC [34]. Instead, CD90^+^ and CK14^+^ have been recently postulated as markers for CSC in UC identifying cells in a basal, less differentiated state [10]. Expression of both markers in this subpopulation was positively correlated. CD90^+^ cells isolated from primary UC were highly tumourigenic in xenograft experiments [10]. Notably, attempts to develop signatures for molecular subtypes of UC [8, 19] revealed an association between CK14 expression and squamous differentiation as well as with poor prognosis. Volkmer et al. [10] further proposed a model of differentiation states in UC characterized by co-expression of surface markers and cytokeratins (Fig. 1a). As characterization of cellular subpopulations isolated from primary tumours is hampered by limited material and tumour heterogeneity, we decided to investigate whether the postulated markers and relationships could be detected in established UCCs and be used to isolate subpopulations. Our results show that the proposed model does not hold in its entirety for the UCC lines that are commonly used as models of the disease.

Initially, we generated an expression profile for markers of cellular morphology, cytokeratins and CD surface markers in a representative panel of 11 UCCs. The expression of EMT marker genes correlated with the morphologic appearance of the cell lines, allowing a categorization of the cell lines into either epithelial or mesenchymal phenotypes. Importantly, UCCs with a mesenchymal phenotype did not express CK14, CK5, or CK20 at detectable levels. In UCCs with an epithelial phenotype, expression of the cytokeratins was heterogeneous in keeping with the heterogeneity observed for primary tissues and xenografts [10]. Generally, CK14 expression was rather restricted, as expected for a marker of CSC, which are thought to constitute a minority among the tumour cells. FACS analysis for the surface markers CD90, CD44, and CD49f also revealed heterogeneity within and among the UCCs. Again, as expected for a putative CSC marker, CD90^+^ cells were rather rare in most cell lines, but were more frequent in some mesenchymal-like cell lines, even though they did not express CK14. As, in contrast to cytokeratins, CD markers were well detectable, neither the postulated correlation between CD90 and CK14 expression, nor the correlation between other surface markers and other cytokeratins exist in mesenchymal phenotype UCCs. Moreover, we did not observe any increase in CK14 expression following enrichment of CD90^+^ cells by sorting or by short-term cisplatin treatment, even in cells with an epithelial phenotype. Immunofluorescence stainings for CD90 and CK14 likewise revealed divergences between the two markers. These observations obviously raise the question of whether UCC have lost the co-expression of CD90 and CK14 during cultivation or whether the association between the markers observed in vivo is only a statistical correlation. In particular*,* the cell type corresponding to the UCCs with a mesenchymal phenotype in vivo remains to be identified. In this respect, changes in cellular morphology towards EMT occur at the invasive tumour front [35], but the mesenchymal phenotype in vitro is stable over many passages [36].

As almost all cells of UCCs and of normal uroepithelial cell cultures appeared broadly positive for CD44 and CD49f, these proteins are unlikely candidates for CSC markers in UC. Urothelial CSCs are likely to be positive for CD44, but further specific markers would be needed for their isolation. Chan et al. [37] reported that 13 out of 14 primary UC were positive for CD44 to various extents and that the CD44^+^ subpopulation displayed increased tumour-initiating capacity in xenografts, but CSCs could not be purified by means of CD44 only. Moreover, CD44 is broadly expressed in the normal urothelium, UC, and even in stromal cells [34], barring its application as a marker on its own for the isolation of UC CSC [38].

To investigate whether CD90 is a reliable marker of CSC in UCCs, we further characterized cellular properties of the subpopulations in the UCCs that were positive for CD90. Initially, we used a magnetic immunoenrichment technique (MACS) to enrich or deplete CD90^+^ cells from UCCs. Upon reculturing these cell fractions, we found that both fractions reconstituted a similar proportion of CD90^+^ and CD90^−^ cells. This finding suggests an active regulation of the distribution of differentiation states in the UCCs, as expected in a tissue hierarchy. Of note, the restoration of the CD90^+^ population from the depleted fraction might originate from the remaining CD90^+^ cells within the depleted fraction due to the impurity of magnetically enriched subpopulations rather than dedifferentiation of CD90^−^ cells. Nevertheless, we did not observe a significant increase in clonogenicity or cisplatin resistance of the MACS-enriched CD90^+^ fraction. Moderately increased clonogenicity and cisplatin resistance was detected in highly purified CD90^+^ cell fractions from FACS sorting. Concurring with these observations, the CD90^+^ fraction was significantly enriched in about half of the UCCs by short-term treatment with cisplatin at IC_50_ concentrations, while the other surface markers remained largely unchanged. These data suggest that CD90^+^ cells might indeed be more resistant to cisplatin and more clonogenic than other fractions in the UCCs, but that the differences are more quantitative than qualitative. Indeed, in cisplatin-resistant sublines generated by long-term treatment, we observed no selection for CD90^+^ cells. In conclusion, CD90 might not constitute a stable marker for cells that persist during cisplatin-based chemotherapy in UC patients and drive recurrence and progression of the disease. Similarly, CK14 neither seems a useful marker in this setting, as cytokeratin expression was rather lost in long-term treated cells which tended towards a more mesenchymal morphology.

LTT-UCCs were established by continuous exposure to increasing cisplatin concentration as we were interested in selection for highly resistant cells and to characterize them rather regarding stem cell properties than comprehensively for resistance mechanisms. Thus, underlying mechanism of acquired resistance might differ from resistant cell lines established by intermittend protocols. However, to our knowledge, to date no conclusive data has been published on resistance acquired by different treatment protocols. Further, our observations were in accordance with cell culture models with acquired resistance from the RCCL [24]. Like docetaxel-resistant prostate cancer cell lines [39], they were slow-cycling, but displayed increased clonogenic capability after treatment with cisplatin compared to parental cells. As observed in many other cancer lines developing resistance to chemotherapeutic drugs, e.g. from head and neck cancers [40], A549 lung cancer cells [41] and ovarian cancer [42], LTT-UCCs appeared to evade cisplatin-induced apoptosis by phenotypic plasticity towards an EMT-like phenotype. We observed morphological changes into spindle-shaped and mesenchymal-looking phenotype and gene expression changes indicative of EMT, including changes in pertinent transcription factors and tight-junction protein genes. The relation between EMT and chemoresistance has been extensively discussed in the context of the hallmarks of cancer and properties of CSC [43–45].

Like acquisition of EMT, activation of WNT-signalling was previously observed in cisplatin resistant non-small cell lung cancer and osteosarcoma cell lines [41, 46]. WNT-signalling regulates cell growth and differentiation and the maintenance of epithelial stem cell compartments in various tissues [47]. Uncontrolled WNT-signalling can lead to constitutive renewal and aberrant expansion of the stem cell or progenitor pool [48, 49]. While WNT-signalling is involved in urothelial regeneration acting on the basal compartment [50], in untreated UCCs WNT-signalling is not generally constitutively activated [25, 51] and its function in UC is generally unresolved. While analysing the changes in intercellular adhesion in LTT-UCC, we discovered some changes in β-catenin intracellular localisation and its increased expression. Typical downstream targets of canonical WNT-signalling like *AXIN-2*, *CCDN1*, *c-MYC*, and *PITX2* were induced, suggesting activation of this pathway subsequent to long-term treatment of UCC with cisplatin. However, a reporter assay for active WNT-signalling revealed only moderate stimulation of activation and a WNT-signalling inhibitor did not revert resistance [52]. We reported already previously that canonical WNT-signalling is not generally constitutively activated in untreated UCCs [25]. Thus, our new results suggest that enhanced expression of typical WNT-target genes could be part of the development of cisplatin resistance in UCC, which might not result from the canonical Wnt-signalling that can be inhibited by niclosamide, but rather from a crosstalk between different pathways.

Despite the canonical WNT-pathway, signalling via β-catenin, WNT can also signal via secondary pathways, as such as protein kinase A and C, calcium-dependent signalling, JNK or Rho-like GTPases. WNT-5a can activate some of those, while inhibiting the canonical WNT-signalling in parallel [53]. Thus, activation of WNT-ligands in the course of resistance development may lead to an upregulation of WNT-target genes in a β-catenin-independent manner. This would explain why we observed changes in WNT-target gene expression in LTT cells, but neither dramatic changes in β-catenin expression nor in the reporter assay. Further, niclosamide, a typical WNT-inhibitor, affected target gene expression in HCT-116 control cells, as expected, but not in UCCs. These results point to the concept that induced expression of WNT-target genes in resistant UCCs might rather originate from a context-dependent crosstalk between different pathways (e.g. WNT, NOTCH, Hedgehog, TGF-β) than from canonical WNT-signalling. Components of the WNT-pathway have been reported to interact with NOTCH components. Likewise the Hedgehog pathway can modulate WNT-signalling, and different components can be shared between WNT- and TGF-β-signalling [54, 55]. This networking of various pathways can lead to an activation of the β-catenin/TCF4 transcription machinery in a non-canonical and alternate manner. As a conclusion, neither differential expression of WNT-target genes in resistant cells nor resistance itself may be reverted by treatment with a WNT-inhibitor only and might rather demand a combined inhibitor treatment strategy.

## Conclusions

In conclusion, the postulated differentiation markers CK14, CK5, and CK20 as well as CD90, CD44, and CD49f were neither sufficient to define a CSC population in UCCs nor to define UCCs with an increased resistance to cisplatin treatment. In addition, cisplatin did not enrich for a UC population defined by these markers, but rather induced phenotypic plasticity likely to be associated with EMT and enhanced the WNT-signalling activity. Thus, in the future LTT-UCCs need to be characterized by expression profiling regarding the mechanisms of cisplatin resistance [56] and further investigated for surface molecules that might serve as appropriate selective markers for the isolation of resistant cancer stem cells.

## Additional files

Additional file 1: Table S1. Primer sequences for quantitative real-time-PCR. Sequences of primers (5′-3′) used for quantitative real-time-PCR including length of PCR product and annealing temperature. *bp* base pair; *Fwd* Forward; *Rev* Reverse. (DOC 48 kb) Additional file 2: Figure S2. Endogenous abundance of subpopulations from differentiation states and subsequent to short-term cisplatin treatment. Original flow cytometry data displaying abundance of CD90, CD44, and CD49f positive cells in 11 UCCs corresponding to the summarized data in Fig. 1d. Unstained cells were used to set gates for positively stained cells. Subsequent to short-term treatment with cisplatin (STT, 72 h) most cell lines displayed increased numbers of CD90+ cells as also illustrated in Fig. 4b; representative results from biological triplicates. STT: Short-term cisplatin treatment. (TIF 4559 kb) Additional file 3: Figure S1. CD90^+^ UCCs do not exhibit a distinct stem cell-like phenotype. Original flow cytometry data for abundance of CD90+ cells corresponding to the summarized data in Fig. 3. a) CD90^+^ fraction in unsorted (top), CD90 magnetically enriched (middle, indicated by arrow) and CD90 depleted (bottom) cell cultures. b) Following reculturing for 7–8 population doublings the number of CD90+ cells was determined again in the respective fractions. (TIF 6628 kb) Additional file 4: Figure S3. Long-term cisplatin treated UCCs are not enriched for CD90^+^/CK14^+^ cells. CD90^+^, CD44^+^, and CD49f^+^ cells in untreated and LTT UCCs as measured by flow cytometry and collectively illustrated in Fig. 5c; representative results from biological triplicates. Unstained cells were used to set gates for positively stained cells. One measurement is shown for each cell line as a representative of biological triplicates. Untr. Ctrl.: Untreated Control; LTT: Long-term cisplatin treatment. (TIF 3017 kb) Additional file 5: Figure S4. Activation of WNT-signalling may contribute to survival of UCCs upon long-term cisplatin treatment. a) Cell viability was measured 72 h after niclosamide treatment by MTT assay in order to determine b) IC_50_ values for RT-112, RT-112-LTT, J82, J82-LTT. HCT-116 cells served as a control. c) Relative RNA expression levels of *β-Catenin*, *AXIN-2*, *CCDN1*, *c-MYC,* and *PITX2* in untreated and niclosamide treated (72 h IC_50_) HCT-116 cells (upper panel) as well as RT-112 and J82 parental cells (lower panel). Expression levels in the untreated control were set as 1. d) Basal activity of a TCF/β-Catenin-dependent promotor after niclosamide treatment in HCT-116 control cells. Mean ± SD of duplicates of TopFlash/FopFlash ratio in niclosamide treated HCT-116 cells and their untreated controls (Untr. Ctrl.). (TIF 1747 kb)

## Abbreviations

CSC: Cancer stem cell
DAPI: 4′,6-diamidino-2-phenylindole
LTT: Long-term cisplatin treatment
SDHA: Succinate Dehydrogenase Complex, Subunit A, Flavoprotein Variant
STT: Short-term cisplatin treatment
TBP: TATA-binding protein
UCC: Urothelial carcinoma cell line
