# Supplementary material for: Phenotype plasticity rather than repopulation from CD90/CK14+ cancer stem cells leads to cisplatin resistance of urothelial carcinoma cell lines
Source: J Exp Clin Cancer Res. 2015 Nov 25;34:144. doi: 10.1186/s13046-015-0259-x (PMC4660687; doi:10.1186/s13046-015-0259-x)
Supplement: Additional file 1: Table S1. — Primer sequences for quantitative real-time-PCR. Sequences of primers (5′-3′) used for quantitative real-time-PCR including length of PCR product and annealing temperature. bp base pair; Fwd Forward; Rev Reverse. (DOC 48 kb) [file 13046_2015_259_MOESM1_ESM.doc]

**Supplementary Material**

Table S1: Primer sequences for quantitative real-time-PCR.

Sequences of primers (5’-3’) used for quantitative real-time-PCR including length of PCR product and annealing temperature. bp.: base pair; Fwd.: Forward; Rev.: Reverse.

| **Gene Name** | **Size [bp]** | **Sequence 5‘-3‘** | | **T _Annealing_ [°C]** |
| --- | --- | --- | --- | --- |
| SDHA | 140 | Fwd. GCCAGGACCTAGAGTTTGTTCA  Rev. CTTTCGCCTTGACTGTTAATGA | | 55 |
| TBP | 119 | Fwd. CAACAGCCTGCCACCTTA  Rev. GAATAGGCTGTGGGGTCAGT | | 55 |
| CK20 | 138 | Fwd. GAACTGAGGTTCAACTAACGGAGC  Rev. TGGCTAACTGGCTGCTGTAACG | | 57 |
| CK14 | 140 | Fwd. GCGCACCATGCAGAACCTG  Rev. CCTCCACGCTGCCAATCATC | | 59 |
| CK5 | 130 | Fwd. GATGATCCAGAGGCTGAGAGC  Rev. CTCGGCCAGCTTGTTCCTG | | 59 |
| CK7 | 102 | Fwd. GAGATCGACAACATCAAGAACCAG  Rev. GCTTGGCACGAGCATCCTTG | | 57 |
| CK13 | 142 | Fwd. CACTCTGTCTAAGACTGACC  Rev. GTGGCATCCATCTCCACG | | 55 |
| E-Cadherin | 171 | Fwd. ACCAGAATAAAGACCAAGTGACCA  Rev. AGCAAGAGCAGCAGAATCAGAAT | | 60 |
| Vimentin | 159 | Fwd. AATGGCTCGTCACCTTCGTGAAT  Rev. CAGATTAGTTTCCCTCAGGTTCAG | | 60 |
| β-Catenin | 127 | Fwd. GTGCTGAAGGTGCTATCTG  Rev. GAGTCCAAAGACAGTTCTG | | 50 |
| ZEB1 | 130 | Fwd. TCACATAAATCAGGAAGAGATC  Rev. CACTGTGAATTCTTAAGTGCTC | | 55 |
| Twist1 | 118 | Fwd. CTCGGACAAGCTGAGCAAGATTCAG  Rev. ACATAGCTGCAGCTTGCCATCTTG | | 60 |
| CLDN3 | 143 | Fwd. TAAGGGACAGACGCAGGGAGAC  Rev. GGGGGCTTCCTGGCTTCT | | 59 |
| CLDN4 | 85 | Fwd. CCAGGATAGCTTAACCCTGACTT  Rev. GCAGACAGAGTGGGGAAAATG | | 59 |
| Axin-2 | 99 | Fwd. CAGCAGCTTCCGTGAGGA  Rev. TTGGTGACCTGGCCCTTG | | 59 |
| CCDN1 | 69 | Fwd. CGCAAACACGCGCAGACCT  Rev. GGAGGGCGGATTGGAAAT | | 60 |
| c-Myc | 94 | Fwd. GCTCCATGAGGAGACACC  Rev. CCTCTTTTCCACAGAAAC | | 55 |
| Pitx2 | 178 | Fwd. TGCGCTCCCTCTTTCTCC  Rev. CGGCAGCGGACTCACTTTAC | | 56 |
| mir200 | | | Hs_miR-200c_1 miScript Primer Assay  Catalogue number MS00003752  Qiagen. Hilden, Germany | |
