# Supplementary figures and images for: Phenotype plasticity rather than repopulation from CD90/CK14+ cancer stem cells leads to cisplatin resistance of urothelial carcinoma cell lines
Source: J Exp Clin Cancer Res. 2015 Nov 25;34:144. doi: 10.1186/s13046-015-0259-x (PMC4660687; doi:10.1186/s13046-015-0259-x)

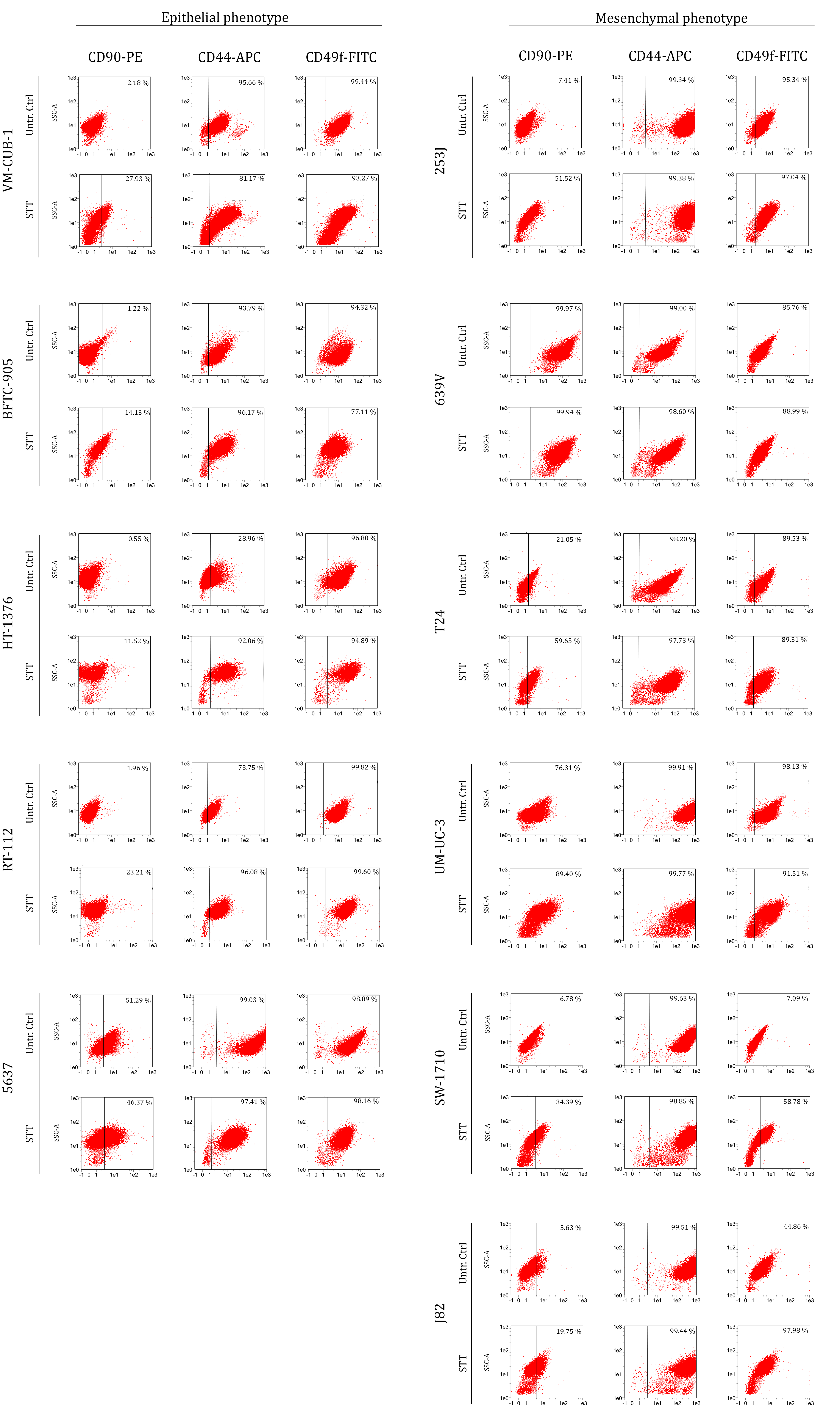

Supplement: Additional file 2: Figure S2. — Endogenous abundance of subpopulations from differentiation states and subsequent to short-term cisplatin treatment. Original flow cytometry data displaying abundance of CD90, CD44, and CD49f positive cells in 11 UCCs corresponding to the summarized data in Fig. 1d. Unstained cells were used to set gates for positively stained cells. Subsequent to short-term treatment with cisplatin (STT, 72 h) most cell lines displayed increased numbers of CD90+ cells as also illustrated in Fig. 4b; representative results from biological triplicates. STT: Short-term cisplatin treatment. (TIF 4559 kb) [file 13046_2015_259_MOESM2_ESM.tif]

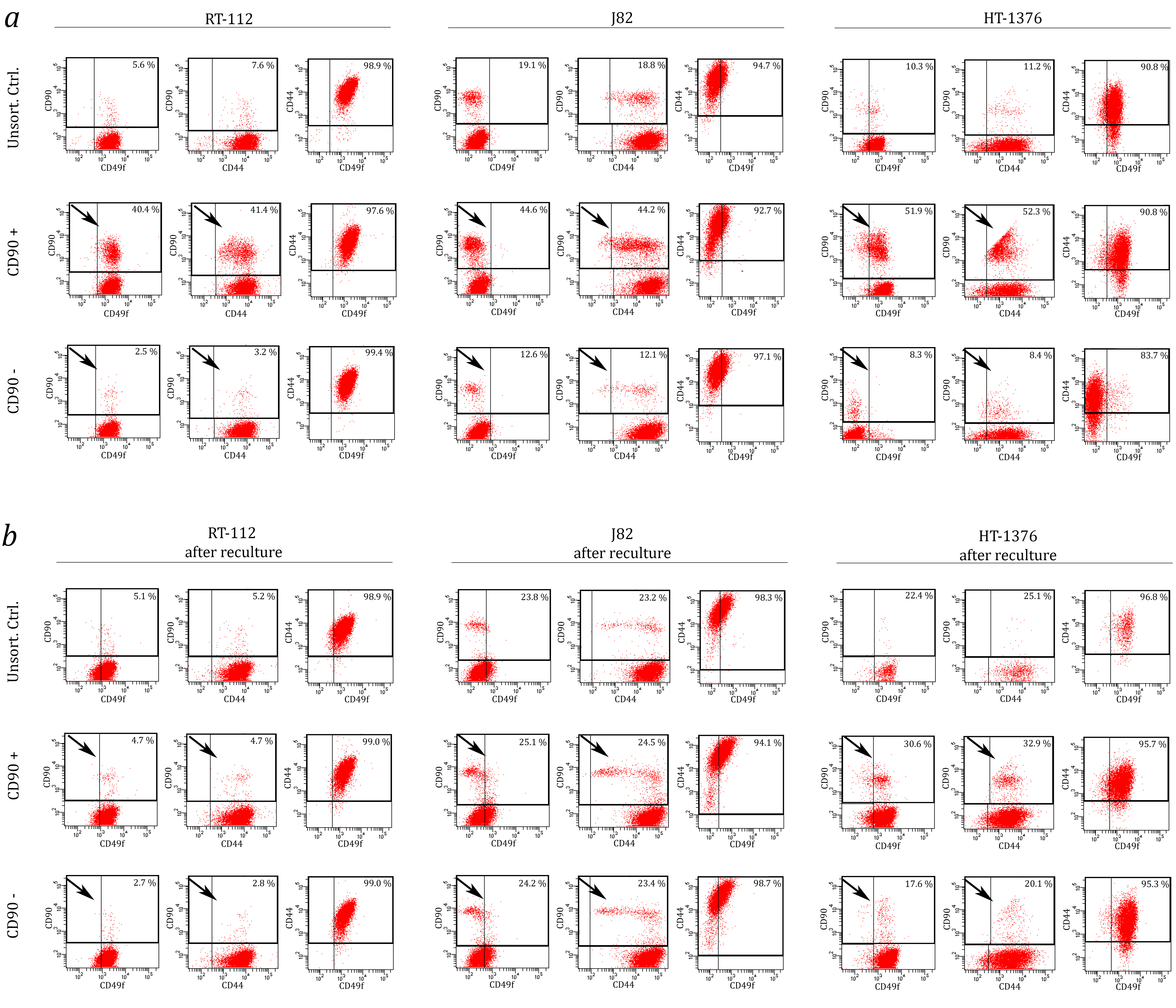

Supplement: Additional file 3: Figure S1. — CD90+ UCCs do not exhibit a distinct stem cell-like phenotype. Original flow cytometry data for abundance of CD90+ cells corresponding to the summarized data in Fig. 3. a) CD90+ fraction in unsorted (top), CD90 magnetically enriched (middle, indicated by arrow) and CD90 depleted (bottom) cell cultures. b) Following reculturing for 7–8 population doublings the number of CD90+ cells was determined again in the respective fractions. (TIF 6628 kb) [file 13046_2015_259_MOESM3_ESM.tif]

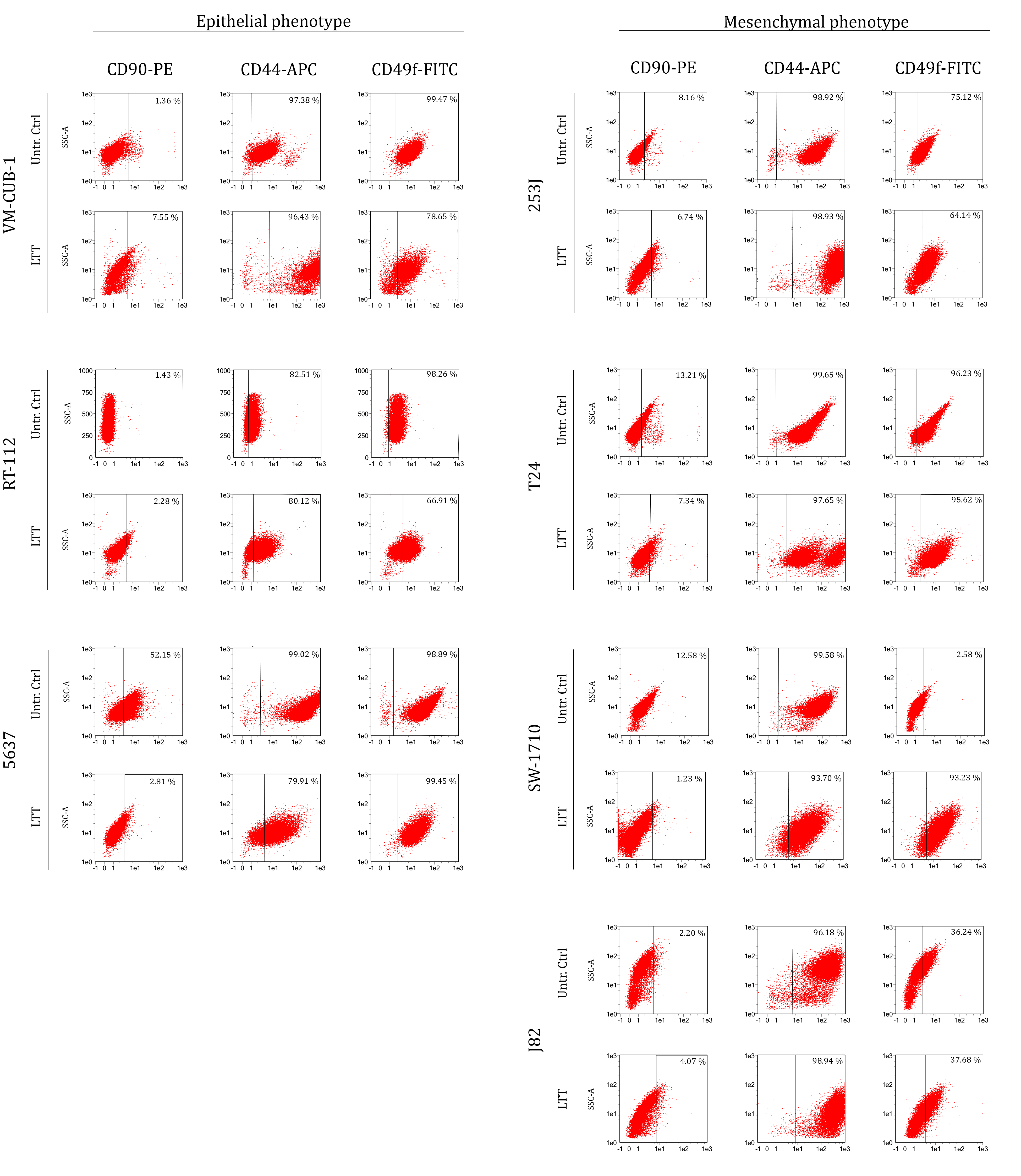

Supplement: Additional file 4: Figure S3. — Long-term cisplatin treated UCCs are not enriched for CD90+/CK14+ cells. CD90+, CD44+, and CD49f+ cells in untreated and LTT UCCs as measured by flow cytometry and collectively illustrated in Fig. 5c; representative results from biological triplicates. Unstained cells were used to set gates for positively stained cells. One measurement is shown for each cell line as a representative of biological triplicates. Untr. Ctrl.: Untreated Control; LTT: Long-term cisplatin treatment. (TIF 3017 kb) [file 13046_2015_259_MOESM4_ESM.tif]

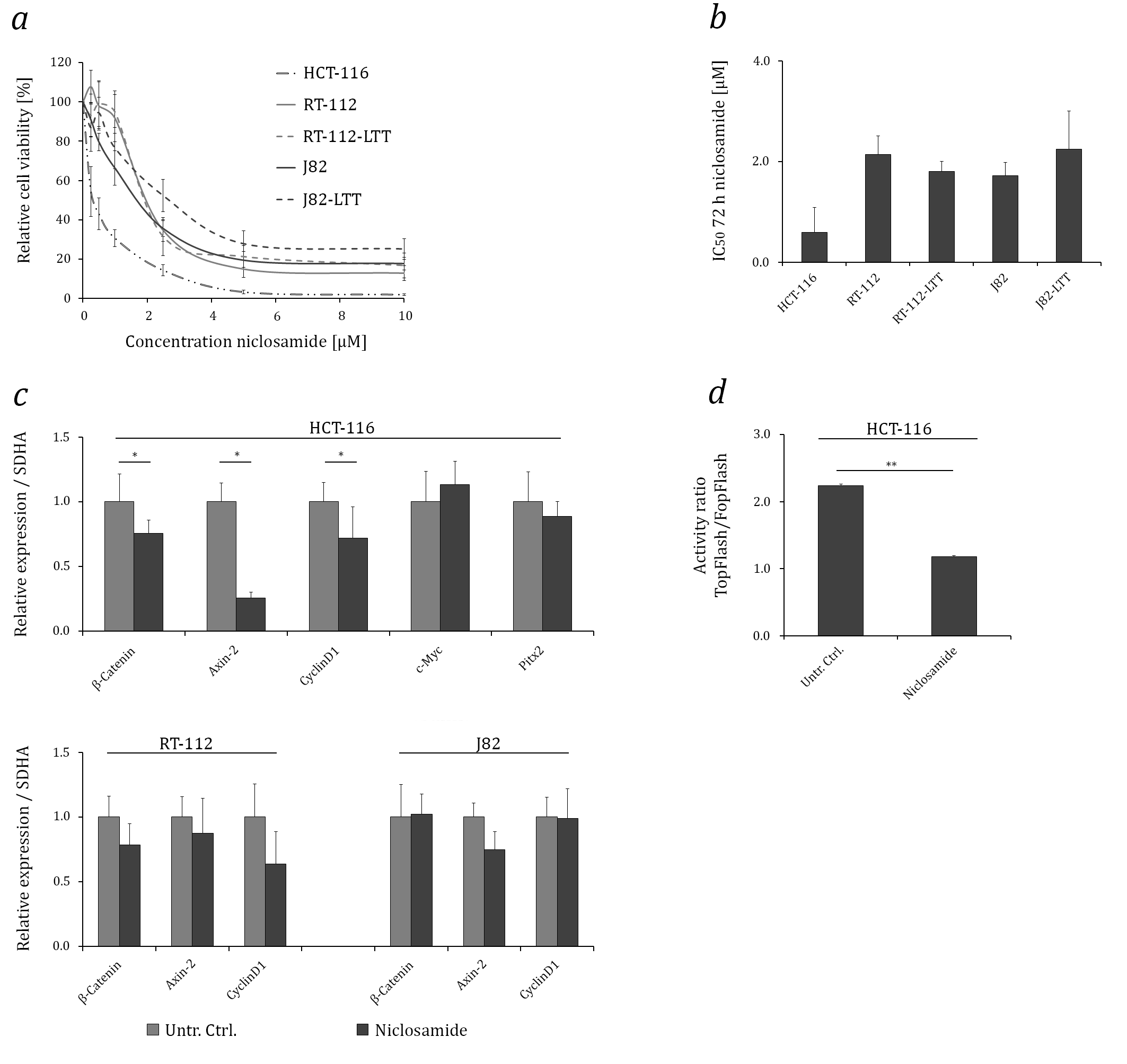

Supplement: Additional file 5: Figure S4. — Activation of WNT-signalling may contribute to survival of UCCs upon long-term cisplatin treatment. a) Cell viability was measured 72 h after niclosamide treatment by MTT assay in order to determine b) IC50 values for RT-112, RT-112-LTT, J82, J82-LTT. HCT-116 cells served as a control. c) Relative RNA expression levels of β-Catenin, AXIN-2, CCDN1, c-MYC, and PITX2 in untreated and niclosamide treated (72 h IC50) HCT-116 cells (upper panel) as well as RT-112 and J82 parental cells (lower panel). Expression levels in the untreated control were set as 1. d) Basal activity of a TCF/β-Catenin-dependent promotor after niclosamide treatment in HCT-116 control cells. Mean ± SD of duplicates of TopFlash/FopFlash ratio in niclosamide treated HCT-116 cells and their untreated controls (Untr. Ctrl.). (TIF 1747 kb) [file 13046_2015_259_MOESM5_ESM.tif]
